# Leucine Supplementation Differently Modulates Branched-Chain Amino Acid Catabolism, Mitochondrial Function and Metabolic Profiles at the Different Stage of Insulin Resistance in Rats on High-Fat Diet

**DOI:** 10.3390/nu9060565

**Published:** 2017-06-02

**Authors:** Rui Liu, Hui Li, Wenjuan Fan, Qiu Jin, Tingting Chao, Yuanjue Wu, Junmei Huang, Liping Hao, Xuefeng Yang

**Affiliations:** Department of Nutrition and Food Hygiene, Hubei Key Laboratory of Food Nutrition and Safety, MOE Key Laboratory of Environment and Health, School of Public Health, Tongji Medical College, Huazhong University of Science and Technology, Wuhan 430030, Hubei, China; amicable123lr@163.com (R.L.); 15032280368@163.com (H.L.); fanwenjuan0706@163.com (W.F.); 15927486696@163.com (Q.J.); weikeccs@sina.com (T.C.); wuyuanjue@hust.edu.cn (Y.W.); huangjunmeihust@163.com (J.H.); haolp@mails.tjmu.edu.cn (L.H.)

**Keywords:** leucine, BCAAs, BCAA catabolism, insulin resistance, metabolomic, mitochondria

## Abstract

The available findings concerning the association between branched-chain amino acids (BCAAs)—particularly leucine—and insulin resistance are conflicting. BCAAs have been proposed to elicit different or even opposite effects, depending on the prevalence of catabolic and anabolic states. We tested the hypothesis that leucine supplementation may exert different effects at different stages of insulin resistance, to provide mechanistic insights into the role of leucine in the progression of insulin resistance. Male Sprague-Dawley rats were fed a normal chow diet, high-fat diet (HFD), HFD supplemented with 1.5% leucine, or HFD with a 20% calorie restriction for 24 or 32 weeks. Leucine supplementation led to abnormal catabolism of BCAA and the incompletely oxidized lipid species that contributed to mitochondrial dysfunction in skeletal muscle in HFD-fed rats in the early stage of insulin resistance (24 weeks). However, leucine supplementation induced no remarkable alternations in BCAA catabolism, but did enhance mitochondrial biogenesis with a concomitant improvement in lipid oxidation and mitochondrial function during the hyperglycaemia stage (32 weeks). These findings suggest that leucine trigger different effects on metabolic signatures at different stages of insulin resistance, and the overall metabolic status of the organisms should be carefully considered to potentiate the benefits of leucine.

## 1. Introduction

Branched-chain amino acids (BCAAs), comprising leucine, isoleucine and valine, are essential amino acids and important nutrient signals that have direct and indirect effects on metabolism. Substantial evidence indicates that increased dietary intake of BCAAs, particularly leucine, has positive effects on the regulation of body weight, muscle protein synthesis, glucose homeostasis, lipid metabolism, and the ageing process [1,2,3,4]. Nevertheless, the idea that BCAAs or their supplementation might have a positive role in glucose metabolism and insulin resistance remains controversial. In other studies, leucine supplementation has no effect, or leads to deterioration in insulin sensitivity [5,6]. Along with these effects on metabolic health, it has long been recognized that elevated levels of circulating BCAAs and related metabolites are strongly associated with obesity and insulin resistance and are predictive of future type 2 diabetes mellitus (T2DM) in humans and in some rodent models [7,8,9,10]. The mechanisms underlying these paradoxical findings are not completely understood.

BCAAs modulate insulin resistance via multiple mechanisms. Generally, BCAAs have been shown to induce insulin resistance by phosphorylation of serine IRS-1 via activating mammalian targets of the rapamycin complex 1 (mTORC1) signaling pathway, and lead to a negative feedback loop of insulin signaling [11]. However, several previous studies, including a study conducted in our laboratory, have indicated that BCAA–associated mTORC1 activation is not required or sufficient to elicit insulin resistance [12,13]. Alternatively, the Lynch group have observed that abnormal tissue-specific BCAA metabolism in obesity results in the accumulation of BCAAs and related toxic metabolites, thereby triggering “anaplerotic stress” and mitochondrial dysfunction associated with insulin resistance and type 2 diabetes mellitus (T2DM) [14,15]. In contrast, BCAAs supplementation has been shown to promote mitochondrial biogenesis and extend the lifespan of yeast and middle-aged mice [16]. Recently, BCAAs and leucine have been hypothesised to elicit different or even opposing effects, depending on the catabolic and anabolic states of the organism [17]. Development of T2DM is a time-dependent process accompanied with different metabolic characteristics [18]. Notably, during the progression of insulin resistance to T2DM, the predominantly anabolic processes transition to predominantly catabolic processes, with the loss of insulin activity. This complexity introduces interpretive limitations, when using data derived from a static time point, on understanding how BCAAs affect the progression of insulin resistance [19].

In the present study, we performed comprehensive metabolic and physiological profiling to investigate dynamic alterations in BCAA catabolism, mitochondrial function, and metabolic responses to long-term leucine supplementation in rats with hyperinsulinaemia or hyperglycaemia induced by a high-fat diet (HFD). We aimed to provide mechanistic insights into the role of leucine in insulin resistance progression. Furthermore, we compared the effects of leucine with those of calorie restriction (CR), a well-known approach for improving insulin sensitivity. 

## 2. Materials and Methods 

### 2.1. Animals, Diets and Treatments 

Male Sprague-Dawley rats at six weeks of age (160–180 g) were obtained from Sino-British Sipper/BK Lab Animal Co., Ltd. (Shanghai, China) and housed in a temperature- and humidity-controlled environment on a 12 h light/dark cycle with free access to food and water. After acclimation, the rats were randomly divided into four groups (*n* = 20 per group) based on body weight and fed a normal chow diet (ND; D12450B, Research Diet Inc., New Brunswick, NJ, USA), HFD (D12451, Research Diet Inc., New Brunswick, NJ, USA), HFD supplemented with 1.5% leucine (HFD + Leu) or HFD with 20% CR (HFD + CR). The administered leucine dose was determined in our preliminary experiments [12] and nearly doubled the total daily leucine intake from food. The diet of the HFD + Leu group was also supplemented with small amounts of valine and isoleucine to prevent valine and isoleucine deficiencies. The HFD was rendered isonitrogenous and isocalorie to the HFD + Leu diet by the addition of a mixture of non-essential amino acids (alanine, glycine, proline, aspartate and serine in isomolar amounts) (Table 1). Food intake was monitored daily, and that of HFD controls was used as a reference to calculate the amount of food provided to the rats subjected to CR (80% of food consumption of controls). Half of the rats in each group were killed at week 24, and the other half were killed at week 32 after overnight fasting. Blood samples were obtained and centrifuged at 4000× *g* for 20 min. The serum was then collected and stored in a freezer at −80 °C until further analysis. Tissues were rapidly excised, weighed, flash-frozen in liquid nitrogen and then stored at −80 °C until use. All animal studies were conducted in accordance with the National Institutes of Health guide for the Care and Use of Laboratory Animals and approved by the Institutional Animal Care and Use Committee of Tongji Medical College, Huazhong University of Science and Technology (IACUC No. 417, Date: 2015.3.28).

### 2.2. Insulin Sensitivity Analysis 

After 24 or 32 weeks on the respective diets, the fasting serum glucose levels were determined by colorimetric assay (Nanjing Jiancheng Bioengineering Institute, Nanjing, China), and insulin levels were assessed using a commercial ELISA kit (Mercodia AB, Uppsala, Sweden), according to the manufacturer’s instructions. The HOMA-IR index was calculated using the following formula: HOMA-IR = [fasting glucose levels (mmol/L)] × [fasting serum insulin (mU/L)]/22.5. The glucose tolerance test (GTT) and insulin tolerance test (ITT) were performed following intragastric glucose administration and intraperitoneal insulin injection, respectively, after overnight starvation of the rats. Blood samples were collected from the tail vein at 0, 15, 30, 60 and 120 min, and the glucose levels were measured with a Glucose Meter (Roche Diagnostics, Shanghai, China). 

### 2.3. Western Blot Analysis

Frozen gastrocnemius muscle, liver and adipose tissues were lysed in RIPA buffer (Beyotime Biotechnology, Shanghai, China) supplemented with phosphatase inhibitors and PMSF (Roche, Ltd., Basel, Switzerland) before use. For the total MitoProfile Oxidative phosphorylation complexes (OXPHOS) analysis, mitochondrial proteins were extracted from the gastrocnemius muscle according to the manufacturer’s instructions (Nanjing Jiancheng Bioengineering Institute, Nanjing, China). Protein concentrations were assayed using a bicinchoninic acid (BCA) kit (Beyotime Biotechnology, Shanghai, China). A total of 20–50 μg of protein were resolved on SDS-PAGE gels and then transferred to polyvinylidene difluoride (PVDF) membranes. Information regarding the primary antibodies used in this study is provided in Table S1. Protein bands were visualized with Molecular Imager VersaDoc MP 4000 System (Bio-Rad, Berkeley, CA, USA).

### 2.4. Transmission Electron Microscopy

The mitochondrial morphology was investigated using electron microscopy. Briefly, gastrocnemius muscles were isolated and fixed with 2.5% glutaraldehyde for 1 day. The muscles were washed and cut longitudinally into 0.5 mm thick strips. Following osmification with 2% osmium tetroxide and 1% uranyl acetate en bloc, the stained tissue was routinely dehydrated in a methanol gradient and embedded in Eponate-12. After polymerization, the ultrastructural features of the mitochondria were observed and photographed using a Tecnai G2 12 transmission electron microscope (FEI Company, Holland, The Netherlands).

### 2.5. Quantitative Real-Time PCR Analysis

Total RNA was extracted from gastrocnemius muscles with Trizol reagent (Invitrogen, New York, NY, USA) and reverse transcribed into cDNA using a high-capacity cDNA archive kit (Takara, Dalian, China). Real-time PCR was performed using the StepOnePlus^TM^ Real-Time PCR System (Applied Biosystems, Grand Island, NY, USA) with the cDNA templates, gene-specific primers and the SYBR Green qPCR Master mix (Takara, Dalian, China). Calculations were performed using a comparative method (2^−ΔΔCt^), with GAPDH as the internal control. Total DNA was extracted from the gastrocnemius muscles using acommercial kit (Tiangen Biotech Co., Ltd., Beijing, China), according to the manufacturer’s instructions. The mitochondrial DNA (mtDNA) content was assessed using RT-PCR by measuring the threshold cycle ratio of a mitochondria-encoded gene (COXII) to a nucleus-encoded gene (β-globin). All RT-PCR experiments were performed in triplicate using the same sample. The primer pairs for each gene are shown in Table S2.

### 2.6. Statistical Analysis

Unless otherwise stated, data are presented as means ± SD. Statistical analyses were performed using the SPSS 15.0 software package (SPSS, Inc., Chicago, IL, USA). Significant differences were assessed by student *t* test or one-way ANOVA followed by the Student-Newman-Keuls test. *p* < 0.05 were considered statistically significant.

### 2.7. Serum Preparation and Metabolite Profiling

A targetedLC-MS/MSbased analysis was performed in the State Key Laboratory of Quality Research in Chinese Medicine at Macau University of Science and Technology and Beijing Mass Spectrometry Medical Research Co., Ltd. Broad metabolite profiling of serum was performed using LC-MS/MS (ACQUITYTM ultra-Performance LC (Waters, Milford, MA, USA) equipped with an AB 4000 Q-TRAP mass spectrometer (Applied Biosystems, Grand Island, NY, USA). Serum (200 μL) was mixed with 200 µL of n-Ethylmaleimide (10 mol/L) in PBS buffer and 1000 µL of methanol containing the internal standard L-phenylalanine-d5 (Phe-d5) at a concentration of 10 ng/mL. The mixture was incubated at −20 °C for 20 min and centrifuged at 12,000 rpm for 10 min at 4 °C. The supernatant was evaporated under a vacuum, and the dry residue was reconstituted with distilled water in preparation for analysis.

In addition to the internal standards used for quality control, a quality control (QC) sample was prepared and analyzed after every 10 serum samples. The blank serum used for QC was prepared from serum that had been stripped of endogenous materials by adding 6 g of charcoal activated powder (Sigma-Aldrich, St. Louis, MO, USA) to 100 mL of serum. This suspension was stirred at room temperature for 2 h and centrifuged at 13,500 rpm for 20 min at 4 °C. Then, the supernatant was filtered using a Millipore Express PES Membrane (Merck Millipore, Ltd., Hesse, Germany). The obtained blank serumwas confirmed to be free of biomarkers using LC-MS/MS.

For quantitative amino acid profiling, the LC-MS/MS analysis was performed on an UltiMate3000 (Dionex, Sunnyvale, CA, USA) equipped with an API 3200 Q TRAP MS System (Applied Biosystems, Grand Island, NY, USA). The samples were thawed, extracted and derivatized before analysis. In detail, after dilution with 80 μL of water, the sample was extracted with 500 μL of a mixture of methanol and acetonitrile (1:9, *v*/*v*). The extraction procedure was performed at −20 °C for 10 min after 2 min of vortexing and 1 min of ultrasonication. Then, the residue was re-dissolved in 100 μL of a mixture of methanol and water (1:1, *v*/*v*) with 1 μg/mL of L-2-chlorophenylalanine, and the same steps were repeated (vortexing, ultrasonication and centrifugation). The supernatant (80 μL) was transferred into the sample vial for the analysis. 

### 2.8. Metabolomics Data Processing and Multivariate Analysis

The pre-processed LC-MS/MS data were log-transformed, and the resulting data were analyzed by orthogonal partial least squares projection to latent structure-discriminant analysis (OPLS-DA) using SIMCA-P version 14.0 (Umetrics, Umea, Sweden). OPLS-DA was also used to identify and rank signature metabolites that discriminated the different groups. Qualities of the OPLS-DA models were assessed by R2 indicative of variation described by all components in the model and by Q2, measuring the model ability to predict class membership.

Variables with a variable importance in the projection (VIP) score >1 were considered relevant for group discrimination. Serum metabolite concentrations in HFD + Leu and HFD + CR rats at different time points were compared with their age-matched HFD controls and significantly changed metabolites were analyzed by One-way ANOVA. *p* value < 0.05 was considered statistically significant. In addition, metabolic pathway interpretation of differential metabolites were performed using the MetaboAnalyst 3.0 and KEGG database.

## 3. Results

### 3.1. Body Weight and Calorie Intake

As expected, HFD-fed rats exhibited a higher body weight than controls. However, HFD-induced weight gain was significantly attenuated by CR or leucine supplementation from 3 or 13 weeks, respectively. The rats supplemented with leucine had significantly higher body weight when compared to CR rat from 7 weeks (Figure S1A). Consistent with our experimental design, the total calorie intake of CR rats was reduced by 20% over the 32-week feeding period compared with that of HFD-fed rats. Leucine addition to the high fat diet did not significantly alter calorie intake across all time points, with the exception of a slight decrease during week 3 (Figure S1B). The average daily leucine intake in ND, HFD, HFD + CR and HFD + Leu-fed rats were 0.68 g, 0.69 g, 0.54 g and 1.28 g, respectively. 

### 3.2. Insulin Sensitivity

**Figure 1:**
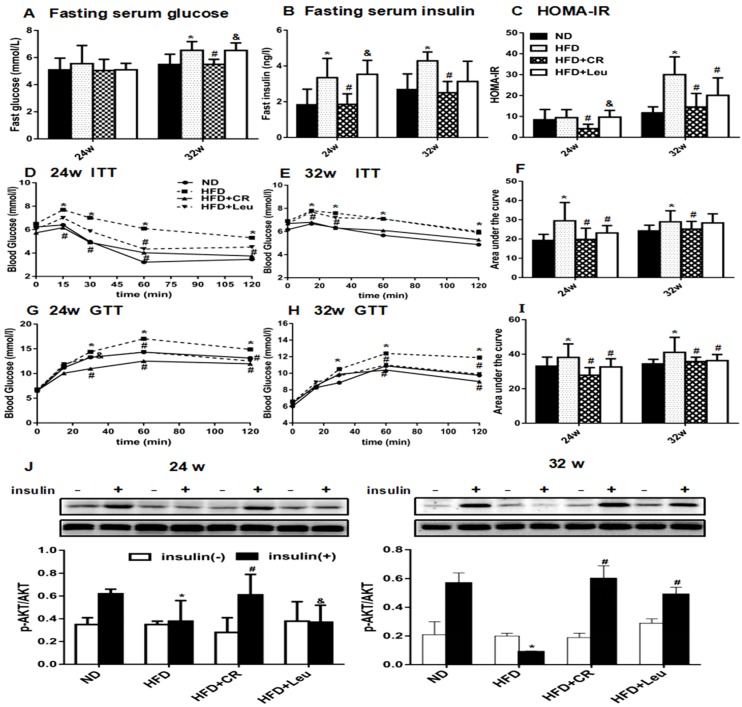
Leucine supplementation improved insulin sensitivity in HFD-fed rats. (**A**) Serum glucose and (**B**) insulin levels were measured after overnight fasting. (**C**) HOMA-IR indices were calculated from fasting glucose and insulin levels. (**D**,**E**) The intraperitoneal ITT (0.8 IU/kg.bw) and (**G**,**H**) OGTT (2 g/kg.bw) were performed after overnight fasting at the end of 24 and 32 weeks, respectively. (**F**,**I**) The area under the curve was calculated (*n* = 8). The results are presented as the mean ± SD. (**J**) AKT and phospho-AKT (Ser473) protein levels in skeletal muscle before (insulin−) and after (insulin+) 2 IU/kg insulin stimulation for 15 min were determined by immunoblotting (*n* = 3–6). * *p* < 0.05 versus ND-fed rats, # *p*< 0.05 versus HFD-fed rats, & *p* < 0.05 versus HFD + CR rats. HFD: High fat diet; ND: Normal chow diet; CR: Calorie restriction; ITT: Insulin tolerance test; OGTT: Oral glucose tolerance test; AKT: Protein kinase B.

As shown in Figure 1A,B, the 24-week HFD did not alter fasting blood glucose but significantly increased fasting serum insulin. Leucine addition to the HFD for 24 weeks had no obvious effects on fasting serum glucose or insulin, but CR prevented the HFD-induced increase in fasting insulin. Accordingly, the HOMA-IR index was unchanged in leucine-supplemented rats, but was decreased in CR rats (Figure 1C). CR rats had lower insulin level and HOMA-IR than HFD + Leu rats at 24 weeks (Figure 1B,C). After 32 weeks, both fasting blood glucose and insulin were elevated in HFD-fed rats compared with ND-fed rats. HFD + Leu diet induced no significant effect on fast serum glucose and insulin levels. CR largely prevented HFD-induced hyperglycaemia and hyperinsulinaemia (Figure 1A,B). The HOMA-IR index was lower in rats treated with either leucine or CR than that in the corresponding HFD-fed controls (Figure 1C), while the CR rats had lower glucose than HFD + Leu rats at 32 weeks (Figure 1A). The results of the OGTT and ITT confirmed that CR or leucine-supplemented rats were significantly more glucose-tolerant and insulin-sensitive than HFD-fed rats (Figure 1D–I) at the two time points. As expected, insulin-stimulated Protein kinase B (PKB/AKT) phosphorylation in the skeletal muscle was increased in CR rats compared with the HFD-fed controls at both time points (Figure 1J). However, leucine-induced AKT phosphorylation was only observed at 32 weeks. The insulin-stimulated AKT phosphorylation protein expression in CR rats was higher than HFD + Leu rats at week 24 (Figure 1J). 

### 3.3. Serum Amino Acid Profiles

Over 24 weeks of feeding, serum concentrations of BCAAs (valine and leucine), glucogenogenic amino acids (alanine, asparagine and glutamate), and gluconeogenic and ketogenic amino acids (phenylalanine and tryptophan) were significantly lower in HFD-fed rats relative to ND control, While the ketogenic amino acids (lysine) was significantly increased (Table 2). However, BCAA (except for isoleucine) concentrations were significantly elevated after leucine supplementation for 24 weeks compared with those in HFD-fed rats. The concentrations of other amino acids, such as aromatic amino acids (phenylalanine and tryptophan) and several gluconeogenic amino acids (asparagine, proline and alanine), were also significantly elevated (Table 3).

At week 32, serum BCAA levels were not significantly changed in HFD rats compared to corresponding controls, but glucogenogenic amino acids (asparagine, cysteine, glutamate and proline), gluconeogenic and ketogenic amino acids (threonine) and ketogenic amino acid lysine were significantly increased (Table 2). However, after 32 weeks of leucine treatment, serum BCAA and aromatic amino acid levels did not significantly differ, whereas asparagine and glycine were significantly reduced and proline was increased. The concentrations of most amino acids in CR rats were reduced at week 24 compared with those in HFD controls, but the observed differences were not statistically significant (except for leucine, isoleucine, alanine, phenylalanine and lysine). However, CR-induced alterations in serum amino acid concentrations were not remarkable at 32 weeks, but alanine and proline were significantly increased (Table 3).

### 3.4. Serum Metabolic Profiles

**Figure 2:**
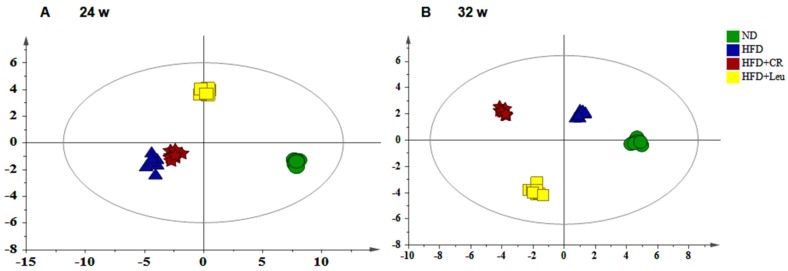
Orthogonal partial least squares projection to latent structure-discriminant analysis (OPLS-DA) score plots displaying the separation of the ND-fed, HFD-fed, Calorie-restricted and HFD + leurats after (**A**) 24 and (**B**) 32 weeks of treatment. Model parameters: (**A**) R2X = 0.944, Q2 = 0.424; (**B**) R2X = 0.938, Q2 = 0.652.HFD: High-fat diet; ND: Normal chow diet.

An LC-MS/MS-based metabolic approach was used to obtain more insights into the mechanisms underlying the different effects of leucine supplementation on insulin resistance. OPLS-DA model was carried out for ND, HFD, HFD + Leu and HFD + CR groups at week 24 and 32, respectively, and the R2 and Q2 values confirmed good qualities for all models (Figure 2A,B). The score plots for each model showed clear separation among the different groups suggesting they had different metabolic features at different pathological stages of HFD induced insulin resistance.

The metabolites detected in the serum were almost decreased in HFD-fed rats compared to the ND controls at week 24. Prominent decreases were observed for metabolites from amino acids including aromatic amino acid catabolites (5-hydroxytryptophan, 4,6-dihydroxyquinoline, and L-kynurenine), methionine catabolites (spermidine and S-adenosylhomocysteine) and nitrogenous compounds derived from amino acid catabolism (creatinine, Creatine, and uridine), fatty acid metabolites (3-hydroxybutyric, Acetylcarnitine and Carnitine), tricarboxylic acid (TCA) cycle intermediates (Fumaric acid and a-Ketoglutaric acid), bile acid metabolites (Ursodeoxycholic acid, Glycochenodeoxycholic acid) and some lysophosphatidylcholines (LPCs). However, fatty acid metabolism (palmitic acid) and TCA intermediate (malic acid) were marked elevated (Table 4). The serum metabolites that discriminated between leucine-supplemented and HFD-fed rats were primarily involved in the TCA cycle, fatty acid, bile acid and amino acid metabolism (Table 5). Specifically, the serum levels ofTCA intermediates (fumaric acid, malic acid and α-ketoglutarate) and metabolites related to lipid metabolism (acylcarnitine, palmitoyl-L-carnitine, carnitine, palmitic acid and 3-hydroxybutyrate) were markedly higher in leucine-treated rats than in HFD-fed controls at week 24. 

The amino acid derivatives (5-hydroxytryptophan, 2-hydroxyisobutyrate, 2-hydroxyglutarate, Creatine and Uridine), fatty acid metabolites (3-hydroxybutyric), TCA cycle intermediates (Malic acid, Citric acid) were significantly elevated after 32 weeks of HFD feeding relative to the age-matched ND control (Table 4). Notably, we observed sharp decreases in the levels of TCA intermediates (fumaric acid, malic acid and citric acid) and lipid-related metabolites (palmitoyl-L-carnitine and palmitic acid) at week 32 relative to HFD-fed controls (Table 5).

In addition, leucine supplementation for 24 weeks contributed to the accumulation of metabolites associated with amino acid catabolism, including aromatic amino acid catabolites (5-hydroxytryptophan, 4,6-dihydroxyquinoline, and L-kynurenine), methionine catabolites (spermidine and S-adenosylhomocysteine) and nitrogenous compounds derived from amino acid catabolism (creatinine, uric acid, and uridine).In contrast, at week 32, the levels of amino acid catabolites that discriminated the leucine group from the HFD-fed controls were reduced. Moreover, prominentincreases in bile acid metabolites and some lysophosphatidylcholines (LPCs) were detected at both 24 and 32 weeks (Table 5).

Metabolites related to lipid metabolism (palmitic acid and LPCs), bile acid metabolism (glycochenodeoxycholic acid and glycocholic acid) and some amino acid catabolites were significantly elevated at week 24 and 32 in CR rats compared with the corresponding HFD-fed controls (Table 5).

### 3.5. BCAA Catabolizing Enzymes and Metabolites

**Figure 3:**
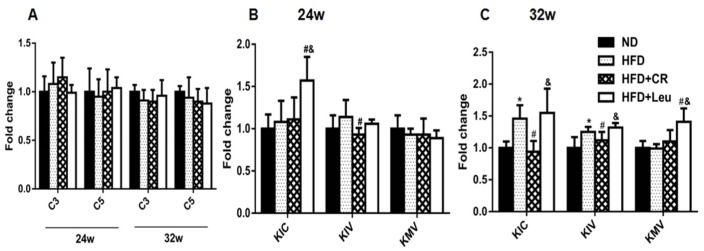
Leucine supplementation-induced alterations in the levels of BCAA derivatives. (**A**) Serum levels of short-chain acylcarnitines (C3 and C5) and (**B**,**C**) Branched-chain α-keto acids (BCKAs) (*n* = 8). The results are presented as means ± SD. * *p* < 0.05 versus ND-fed rats, # *p* < 0.05 versus HFD-fed rats, & *p* < 0.05 versus HFD + CR rats. HFD: High fat diet; ND: Normal chow diet; CR: Calorie restriction.

**Figure 4:**
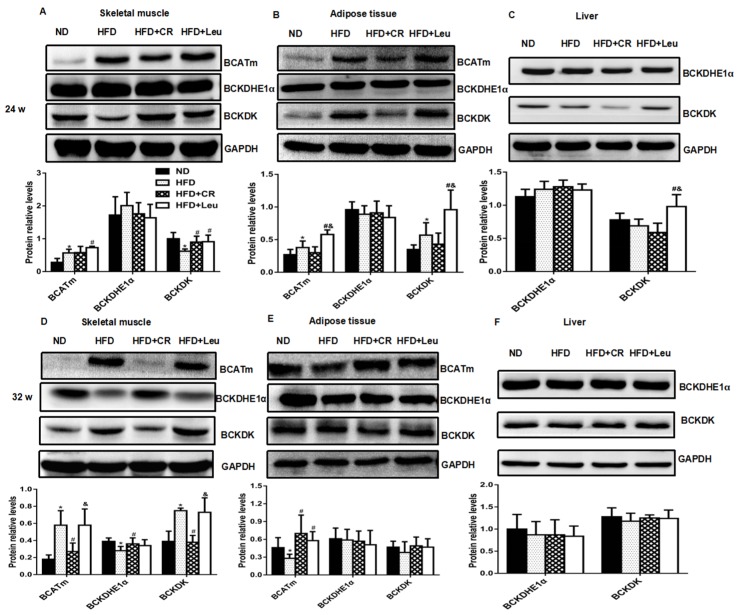
Leucine supplementation-induced alterations in BCAA catabolic enzyme protein expression. Representative immunoblots of total mBCAT, BCKDHE1α and BCKDK protein levels in skeletal muscle (**A**,**D**), adipose tissue (**B**,**E**) and liver (**C**,**F**); *n* = 6. The results are presented as the mean ± SD.* *p* < 0.05 versus ND-fed rats, # *p* < 0.05 versus HFD-fed rats, & *p* < 0.05 versus HFD + CR rats. mBCAT: Mitochondrial branched-chain aminotransferase; BCKDHE1α: Branched-chain α-keto acid dehydrogenase E1 α; BCKDK: Branched-chain α-keto acid dehydrogenase kinase, HFD: High fat diet; ND: Normal chow diet; CR: Calorie restriction.

HFD feeding for 24 weeks, the levels of transamination products of BCAAs— branched-chain α-keto acids (BCKAs)—did not change significantly compared with ND-fed control at 24 weeks (Figure 3B). Mitochondrial branched-chain aminotransferase (BCATm) protein expression in skeletal muscle and adipose tissue were both significantly increased. Branched-chain α-keto acid dehydrogenase kinase (BCKDK) protein expression in HFD rats was lower in skeletal muscle, while higher in adipose tissue than ND controls (Figure 4A–C). BCATm protein expression was significantly increased in skeletal muscle and adipose tissue, concurrent with a significant increase in BCKDKandno change in branched-chain α-keto acid dehydrogenase E1 α (BCKDHE1α) in skeletal muscles, livers and adipose tissues of leucine-supplemented rats, compared with those of HFD-fed controls at week 24 (Figure 4A–C). Furthermore, the levels of transamination products of leucine (α-Ketoisocaproate, KIC) were also increased (Figure 3B). 

However, HFD feeding for 32 weeks led to significantly increase in the levels of transamination products of leucine (α-Ketoisocaproate, KIC) and transamination products of valine (α-Ketoisovalerate, KIV) compared to corresponding ND control (Figure 3C). BCATm and BCKDK protein expression were significantly elevated, but BCKDHE1αprotein expression was significantly decreased in skeletal muscle. In addition, BCATm protein expression in adipose tissue was significantly decreased compared with ND-fed control at 32 weeks (Figure 4D–F). In contrast, the only changes observed in the levels of BCAA catabolizing enzymes and metabolites were an increased expression of BCATm in adipose tissue (Figure 4D–F) and increased isoleucine-derived KMV (α-Keto-β-methylvalerate, KMV) levels compared to the HFD-fed controls after 32 weeks of leucine supplementation (Figure 3C). BCAA-catabolizing enzyme protein expression did not significantly differ between CR rats and HFD-fed controls at week 24 (Figure 4A–C). However, BCATm and BCKDK protein expression was significantly decreased, whereas BCKDHE1α expression was significantly increased in skeletal muscle in CR rats at week 32 compared to the HFD-fed controls (Figure 4D–F). Notably, neither leucine supplementation nor CR had a significant impact on serum BCAA-derived short-chain acylcarnitine (C3 and C5) levels throughout the experiment (Figure 3A). 

### 3.6. Mitochondrial Properties

**Figure 5:**
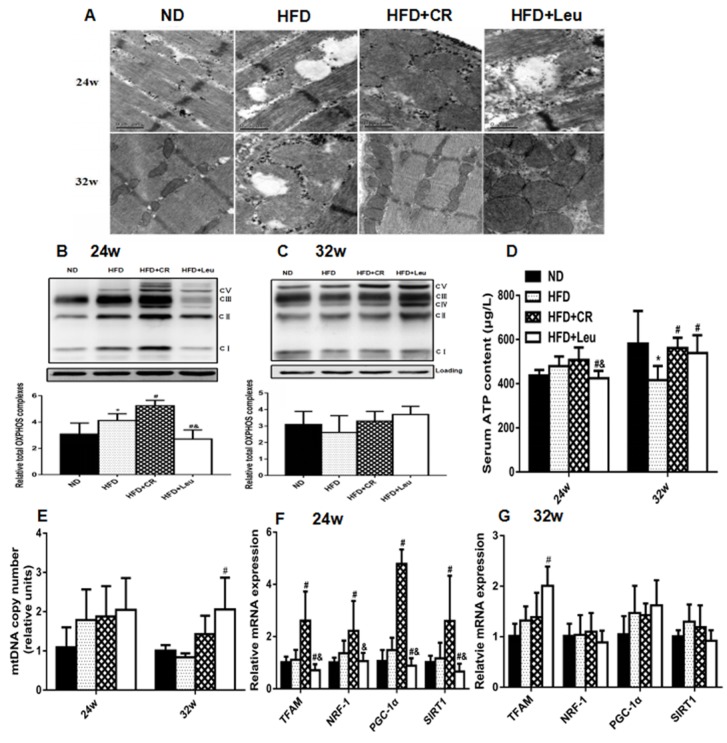
Leucine supplementation-induced alterations in mitochondrial properties. (**A**) Transmission electronic microscopy (×20,000) of the mitochondrial ultrastructure in skeletal muscle (*n* = 4). (**B**,**C**) Protein expression of Oxidative phosphorylation complexes (OXPHOS) complexes I to V in the electron transport chain in skeletal muscle were analyzed by western blotting (*n* = 6). (**D**) Serum ATP levels (*n* = 8). (**E**) Mitochondrial DNA (mtDNA) copy number (*n* = 6). (**F**,**G**) Expression of mitochondrial biogenesis-related genes (*n* = 6). The results are presented as the mean ± SD. * *p* < 0.05 versus ND-fed rats, # *p* < 0.05 versus HFD-fed rats, & *p* < 0.05 versus HFD + CR rats. ND: Normal chow diet; HFD: High fat diet; CR: Calorie restriction.

Long-term HFD feeding-induced mitochondrial structural damage was exacerbated by leucine supplementation at 24 weeks (Figure 5A). Mitochondrial function was also impaired, as indicated by the reduced expression of enzymes involved in OXPHOSand serum ATP production (Figure 5B,D). The expression of genes controlling mitochondrial biogenesis, including mitochondrial transcription factor A (TFAM), peroxisome proliferator-activated receptor γ coactivator-1-α (PGC-1α) and sirtuin 1 (SIRT1), were greatly decreased (Figure 5F) in HFD + Leurats at this time point. In contrast, HFD-induced mitochondrial damage was partially restored by leucine supplementation at 32 weeks (Figure 5A), accompanied by increases in the mtDNA content (Figure 5E), the expression of genes involved in mitochondrial biogenesis (TFAM) (Figure 5G) and ATP production (Figure 5D). As expected, CR ameliorated HFD-induced mitochondrial damage at both time points. At week 24, CR enhanced expression of enzymes involved in OXPHOS, and the gene expression related to mitochondrial biogenesis (TFAM, NRF-1, PGC-1α and SIRT1) (Figure 5B,F). At week 32, CR-induced mitochondrial biogenesis was not greatly changed, but ATP production was significantly increasedcompared to HFD controls (Figure 5D,E,G). CR exhibited better effects on improving HFD-induced mitochondrial damage at week 24 as demonstrated by increases in OXPHOS protein levels (Figure 5B), ATP production (Figure 5D), and mitochondrial biogenesis compared to leucine supplementation (Figure 5F).

## 4. Discussion and Conclusions

The effects of BCAA (especially leucine) supplementation on insulin resistance remains elusive. Numerous studies have shown that leucine supplementation may prevent HFD-induced obesity [1,2,20], modulate glucose metabolism [21,22] and improve insulin sensitivity in rodent models. However, Newgard et al. suggested that excess BCAAs could contribute to development of insulin resistance, at least under conditions of high fat feeding or elevated tissue fatty acid availability [7]. The underlying mechanisms contributing to these inconsistent results are incompletely understood. Exactly how leucine exerts effects on insulin resistance is complex, and may depend on the study design, dietary composition, the animal models typically used and the BCAA concentrations, duration and form of administration. Recently, it has been supposed that BCAAs can trigger different and even opposite effects, depending on the catabolic and anabolic state of the organisms [17]. Given the different metabolic signature in the progression of T2DM, and leucine’s ability to stimulate both anabolic and catabolic processes [23], we applied an integrative, time-resolved approach to gain a deeper understanding the effects of leucine supplementation in the complexity of the disease on BCAA metabolism, mitochondrial properties and metabolic profiling. Several interesting and important findings were obtained.

In the present study, HFD feeding induced hyperinsulinaemia at 24 weeks. During this period, the rats became increasingly obese, and exhibited lower concentrations of amino acids and related metabolites, fatty acid metabolites and TCA intermediates compared to corresponding ND controls. Studies have shown that insulin signaling functions at an increased basal level in the presence of insulin resistance and hyperinsulinaemia [24,25]. Compensatory increased insulin secretion response to overnutrition may slow fat oxidation and promote anabolism (syntheses of glycogen, proteins, and fats), leading to body weight gain and glucose homeostasis. In contrast, after HFD feeding for 32 weeks, the rats progressively developed hyperglycaemia. The increased insulin level could not maintain glucose at normal levels, and was accompanied by elevated levels of amino acids and related metabolites, fatty acid metabolites and TCA intermediates relative to corresponding ND-fed rats. These data suggested that at this stage the anabolic response to hyperinsulinaemia was blunted, HFD rats were transition from predominantly anabolic condition to predominantly catabolic condition.

The effect of supplementation of HFD with leucine was examined in these two different metabolic conditions, respectively. Our results indicated that doubling dietary leucine globally improved HFD-induced insulin resistance but caused different changes in BCAA catabolism in rats with hyperinsulinaemia or hyperglycaemia. These results seem inconsistent with the finding that elevated circulation of BCAAs and related metabolites promotes insulin resistance, particularly in response to HFD feeding [5,7]. Leucine supplementation ameliorated HFD-induced insulin resistance to different extents at the two time points, as indicated by the improved HOMA-IR values, insulin tolerance and glucose tolerance. Interestingly, this leucine-induced elevation in insulin sensitivity may not be correlated with changes in serum BCAAs, which were increased in HFD + Leu rats at 24 weeks and unaltered at 32 weeks compared with those in HFD-fed rats. 

Recent metabolomic, proteomic and genomic studies have suggested that altered BCAA catabolism contributes to elevated BCAAs [14,26,27]. The enzymes involved in the first two steps of the BCAA catabolic pathway, BCATm and Branched-chain α-keto acid dehydrogenase (BCKDH), were examined in the present study. Leucine supplementation increased BCATm protein expression in adipose tissue and skeletal muscle at 24 weeks, suggesting a substrate-induced effect of increased dietary BCAAs (~70% increase for three BCAAs) compared with that in HFD-fed rats. No differences were observed in protein expression of BCKDHE1α, whereas that of BCKDK, a kinase that inactivates BCKDH by phosphorylating the subunit E1α, was increased in observed tissues, including skeletal muscle, liver and adipose tissues. BCKDK participates in a key mechanism for the nutritional and hormonal regulation of BCAA oxidative flux [28,29], and its expression is increased by insulin [30]. This potentially impaired BCKD activity may contribute to the accumulation of BCKAs, such as KIC, in leucine-supplemented rats during the early stage of insulin resistance, which was characterized by hyperinsulinaemia in the present study. Following the progression of insulin resistance to hyperglycaemia, no significant leucine-induced alterations were observed in serum BCAAs or BCAA-catabolizing enzyme levels, with the exception of increased BCATm in adipose tissue. This tight regulation of BCAA catabolism suggests that an elegant method exists for increasing the degradation of BCAAs when they are present in excess and sparing them [31] when they are needed for anabolic or other necessary processes during the different stages of insulin resistance.

In addition to alterations in BCAA catabolism, we identified several leucine-associated changes in TCA cycle intermediates, lipids and other amino acid metabolites in rats with hyperinsulinaemia or hyperglycaemia. Leucine-induced alterations in serum amino acid concentrations at 24 weeks included increased aromatic amino acid (phenylalanine and tryptophan), alanine and asparagine levels, which may have occurred because BCAAs share a competitive transport system with aromatic amino acids [32]. In addition, BCAA transamination provides a nitrogen source for the synthesis of other dispensable amino acids. The levels of almost all amino acid catabolites investigated in the present study were increased in leucine-supplemented rats at 24 weeks, but were unaltered or decreased at 32 weeks, compared with those in HFD-fed controls. It is noteworthy that various factors affect serum amino acid concentrations, including protein turnover (protein synthesis and degradation), and the metabolism of individual amino acids. The exact mechanisms by which leucine supplementation affect the levels of amino acids and their metabolites at different stage of insulin resistance are still unknown, and warrant further study.

Acylcarnitines are intermediates of fatty acid oxidation, and accumulate as a consequence of the metabolic dysfunction resulting from the insufficient integration between β-oxidation and the TCA cycle [33,34,35]. Elevated BCAAs in a lipotoxic environment have been proposed to induce the accumulation of BCAA-derived acylcarnitines and incompletely oxidized lipid species [5,7]. In HFD-induced hyperinsulinaemic rats in the present study, leucine supplementation caused increases in acylcarnitine species (carnitine, acylcarnitine and palmitoyl-L-carnitine), but no remarkable changes in BCAA-derived short-chain acylcarnitines (C3 and C5). However, TCA cycle intermediates were significantly elevated, reflecting an early compensatory response to substrate overload. Accumulated by-products of BCAA transamination are catabolized to BCAA-related acetyl-CoAs, ketones and other intermediates, leading to competitive inhibition of lipid-derived acetyl-CoA entry into the TCA cycle [36,37,38]; in turn, this process may reduce fatty acid oxidation and induce acylcarnitine accumulation and ketogenesis, as indicated by the results (Table 3). Interestingly, as insulin resistance progressed, leucine promoted reductions in the accumulation of serum acylcarnitine species and levels of multiple TCA cycle intermediates at 32 weeks compared with those in age-matched HFD-fed controls.Skeletal muscle serves as a major reservoir of free carnitine and is thought to be a principal contributor to the serum acylcarnitine pool. The accumulation of acylcarnitine species is mitotoxic and promotes mitochondrial dysfunction. The above-mentioned alterations in the metabolic phenotype might be correlated with changes in skeletal muscle mitochondrial properties. Consistent with this hypothesis, the HFD + Leu treatment caused increased mitochondrial damage, accompanied by decreases in mitochondrial biogenesis, ATP production and mitochondrial oxidative enzyme levels in skeletal muscle compared with those in HFD-fed controls.These data were seemingly in conflict with the favorable effects of leucine on overall insulin sensitivity at this stage. It is well-known that insulin resistance is associated with mitochondrial dysfunction, but the causality of this association is controversial. More recent findings suggest that mitochondrial dysfunction is not an early event in the development of insulin resistance [39], but rather an adaptation to excess nutrients [40,41]. Skeletal muscle is the initial site for most BCAA catabolism and major insulin-target tissue. Thus, at this stage, improved insulin sensitivity by leucine supplementation may facilitate both the anabolic effects of insulin and the adaptive catabolic effects of “nutrient overload” in skeletal muscle under the background of a HFD, leading to an impaired mitochondrial function. Notably, the HFD-induced mitochondrial damage was relieved and mitochondrial biogenesis was increased by leucine supplementation after 32 weeks. These results are consistent with previous studies reporting that leucine supplementation increases mitochondrial biogenesis while simultaneously enhancing lipid oxidation [42,43,44], leading to the reduced accumulation of acylcarnitine species. Therefore, we speculate that BCAAs are primarily used for anabolism at this stage; thus, BCAAs and their derivatives that flow into the mitochondria are decreased. This hypothesis is consistent with our observations of decreased levels of TCA cycle intermediates and no obvious changes in BCAAs and amino acids related catabolites concentrations in circulation. The reduced mitochondrial load occurred simultaneously with improved mitochondrial function, leading to increased ATP production. Thus, altered mitochondrial function may partly account for the improvement in skeletal muscle insulin sensitivity. Moreover, Li et al. observed that in HFD-induced obese mice, leucine supplementation prevented obesity and insulin resistance in association with attenuation of mitochondrial dysfunction [45].

**Figure 6:**
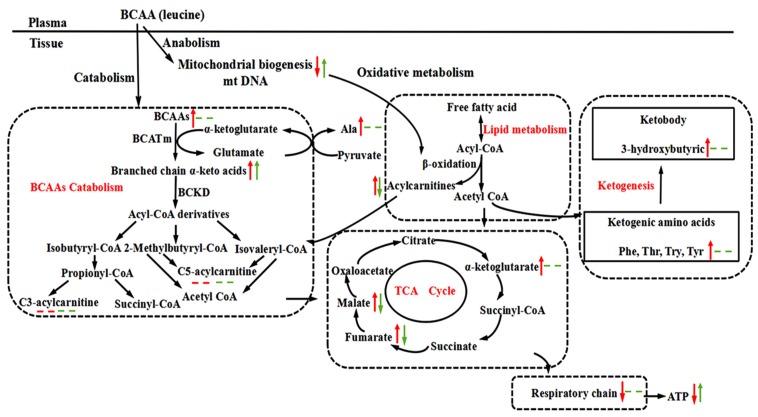
Summary of the possible metabolic alterations induced by leucine supplementation at the different stage of insulin resistance in high-fat diet-fed rats. The red arrows indicate the changes observed at 24 weeks, whereas the blue arrows represent the changes observed at 32 weeks compared with the age-matched high-fat diet-fed controls. The up-arrows indicate an increase in concentration; the down-arrows indicate a decrease in concentration; the dotted lines indicate no significant change in concentration.

Taken together, these results indicated that abnormal BCAA metabolism induced by dietary supplementation coupled with HFD intake might lead to the accumulation of BCKAs and incompletely oxidized lipid species, which contributed to mitochondrial dysfunction in skeletal muscle during the early stage of insulin resistance (24 weeks). In contrast, during the hyperglycaemic stage (32 weeks), when catabolic processes predominate, leucine supplementation enhanced mitochondrial biogenesis, with concomitantly improved lipid oxidation and mitochondrial function in skeletal muscle (Figure 6). 

CR without malnutrition has long been considered an effective strategy for protecting againstage-associated disorders by modulating mitochondrial biogenesis and activating the reactive oxygen species defense system [46,47,48]. However, long-term CR is hard for compliance and its possible disadvantages remain to be determined, particularly in the elderly [49]. There has been a growing interest in developing dietary strategies that mimic the beneficial effects of CR. Dietary supplementation with BCAAs has been shown to improve mitochondrial biogenensis and prevent oxidative damage in middle-aged mice [4,16]. In this study, both CR and leucine supplementation improved HFD-induced insulin resistance, but they differently modulated mitochondrial properties at the different stage of insulin resistance. At the hyperinsulinaemia stage, CR improved skeletal muscle mitochondrial biogenesis and function in HFD rats. However, leucine supplementation increased metabolic overload in mitochondria of skeletal muscle accompanied by impaired mitochondrial biogenesis and function. Interestingly, at the hyperglycaemic stage, both CR and leucine supplementationalleviate the HFD-induced mitochondrial damage, whereas HFD + Leu showed a more pronounced effect on mitochondrial biogenesis than CR. 

Our study has several limitations. First, T2DM progression and development are long-term processes, with different metabolic characteristics at different stages. Our animal studies only revealed alterations at two time points; thus, they may not reflect the total metabolic changes associated with leucine supplementation. Second, the metabolites detected in our study were limited and we only observed the skeletal muscle’s response to leucine supplementation, further investigation should use untargeted metabolomic analysis to capture a large number of differences in metabolite levels and metabolic responses in multiple organs to improve our understanding of the relevant mechanisms. Third, our data demonstrated that the effects of leucine supplementation on insulin resistance were associated with the metabolic condition of the organisms, but the metabolic conditions of HFD rats in the progression of insulin resistance were mainly estimated by the serum metabolites in the present study;this should be further verified by other experiments. 

To our knowledge, this study is the first to report that leucine exerts different effects during different stages of insulin resistance in a diet-induced animal model, depending on the prevalence of catabolic and anabolic signals in the organism [17]. During the early stage of insulin resistance, increased insulin levels to maintain normal blood glucose by promoting anabolism (glycogen, protein and fat synthesis) and suppressing fat oxidation [50,51,52]. Thus, strategies that improve insulin sensitivity may facilitate both the anabolic effects of insulin and the adaptive catabolic effects of “nutrient overload” in the context of a HFD, leading to an adverse metabolic phenotype and mitochondrial dysfunction. Leucine supplementation may not be beneficial if calorie intake is not restricted at this stage. However, as insulin resistance progresses and insulin loses its effectiveness, the condition is characterized by a catabolic state. BCAA or leucine supplementation improves insulin sensitivity, mitochondrial function and other metabolic outcomes, as demonstrated in many conditions, including muscle sarcopaenia, burns, trauma and T2DM in elderly patients [53,54,55]. The findings of this study help explain the conflicting results reported by different studies and indicate that the patients’ overall metabolic status should be carefully considered to potentiate the health benefits of leucine or BCAAs in each clinical setting.

## Figures and Tables

**Table 1:** Composition of the experimental diets (/100g).

	ND	HFD	HFD + Leu
**Macronutrients (g)**			
Protein	20.3	20.3	20.3
Carbohydrate	64.5	44.6	44.6
Fat	4.5	24.4	24.4
Fiber	5.0	5.0	5.0
Minerals	3.0	3.0	3.0
Calories (kcal)	379.7	479.2	479.2
Fat (energy %)	10.7	45.8	45.8
Protein (energy %)	21.3	17.0	17.0
Carbohydrate (energy %)	68.0	37.2	37.2
**Amino acids (g)**			
Leucine	1.3	1.5	2.9
Isoleucine	0.6	0.7	1.0
Valine	0.7	0.9	1.1
Alanine	0.8	0.8	0.5
Aspartic acid	1.5	1.6	1.2
Glycine	0.7	0.5	0.3
Threonine	0.7	0.7	0.7
Serine	0.8	1.0	0.8
Glutamic acid	3.4	3.2	3.2
Tyrosine	0.5	0.7	0.6
Phenylalanine	0.7	0.8	0.8
Lysine	1.1	1.2	1.2
Histidine	0.5	0.5	0.4
Proline	1.2	1.7	1.6
Arginine	1.0	0.5	0.6
Methionine	0.4	0.3	0.4
Serine	0.8	1.0	0.8

ND: Normal chow diet; HFD: High fat diet.

**Table 2:** Amino acids profile in HFD rats.

Parameter	24 weeks	32 weeks
BCAAs		
isoleucine	1.01 (0.93, 1.09)	0.97 (0.89, 1.06)
valine	0.62 (0.55, 0.70) *	1.04 (0.96, 1,11)
leucine	0.70 (0.56, 0.84) *	0.92 (0.84, 1.01)
**Glucogenogenic amino acids**		
alanine	0.48 (0.37, 0.59) *	1.02 (0.89, 1.16)
asparagine	0.72 (0.65, 0.80) *	1.36 (1.14, 1.59) *
aspartic acid	1.01 (0.60, 1.43)	1.01 (0.83, 1.20)
cysteine	1.06 (0.92, 1.20)	1.33 (1.24, 1.42) *
glutamate	0.79 (0.69, 0.90) *	1.65 (1.38, 1.92) *
glutamine	1.04 (0.98, 1.10)	1.08 (1.02, 1.14)
glycine	1.46 (1.24, 1.68)	0.94 (0.83, 1.05)
histidine	1.02 (0.95, 1.09)	0.83 (0.77, 0.88) *
methionine	1.01 (0.93, 1.08)	1.09 (0.94, 1.24)
proline	0.91 (0.84, 0.99)	1.23 (1.06, 1.41) *
serine	1.23 (1.04, 1.42)	1.08 (1.02, 1.15)
arginine	1.19 (1.06, 1.32)	0.95 (0.83, 1.08)
**Gluconeogenic and ketogenic amino acids**		
phenylalanine	0.54 (0.48, 0.59) *	1.10 (0.94, 1.25)
threonine	1.05 (0.95, 1.15)	1.34 (1.25, 1.42) *
tryptophan	0.76 (0.68, 0.85) *	0.90 (0.83, 0.98)
tyrosine	1.12 (0.95, 1.29)	0.89 (0.82, 0.96)
**Ketogenic amino acids**		
lysine	1.37 (1.20, 1.54) *	1.46 (1.21, 1.71) *

Data were expressed as mean fold difference (95% CI) of HFD-fed rats relative to their corresponding ND-fed controls at week 24 and 32, respectively (*n* = 8). * *p* < 0.05 versus age-matched ND controls. HFD: High-fat diet; ND: Normal chow diet; CR: Calorie restriction; BCAAs, branched-chain amino acids.

**Table 3:** Amino acids profile.

	24 weeks		32 weeks	
Parameter	CR	HFD + Leu	CR	HFD + Leu
BCAAs				
isoleucine	0.82 (0.76, 0.88) *	0.91 (0.84, 0.98)	0.88 (0.79, 0.96)	0.94 (0.77, 1.11)
valine	0.91 (0.86, 0.96)	1.38 (1.18, 1.58) *	0.94 (0.85, 1.03)	0.95 (0.74, 1.15)
leucine	0.85 (0.79, 0.91) *	1.43 (1.02, 1.84) *	0.91 (0.84, 0.98)	0.98 (0.69, 1.27)
**Glucogenogenic amino acids**				
alanine	0.79 (0.71, 0.87) *	1.49 (1.34, 1.65) *	1.29 (1.02, 1.56) *	0.92 (0.71, 1.12)
asparagine	0.91 (0.76, 1.06)	1.49 (1.25, 1.72) *	0.90 (0.73, 1.06)	0.79 (0.60, 0.98) *
aspartic acid	0.93 (0.82, 1.03)	0.91 (0.74, 1.08)	1.08 (0.96, 1.20)	1.14 (0.97, 1.31)
cysteine	1.19 (1.08, 1.30) *	0.96 (0.85, 1.08)	1.09 (0.93, 1.25)	0.93 (0.77, 1.08)
glutamate	0.93 (0.80, 1.07)	1.07 (0.92, 1.23)	1.08 (0.86, 1.31)	1.00 (0.83, 1.17)
glutamine	0.93 (0.85, 1.01)	0.92 (0.84, 1.00)	1.03 (0.88, 1.18)	1.06 (0.91, 1.21)
glycine	0.96 (0.90, 1.03)	0.95 (0.91, 0.99)	0.89 (0.81, 0.98)	0.82 (0.71, 0.93) *
histidine	0.98 (0.90, 1.05)	0.97 (0.83, 1.11)	1.09 (0.98, 1.20)	1.05 (0.92, 1.18)
methionine	0.94 (0.86, 1.03)	1.01 (0.94, 1.08)	0.94 (0.86, 1.02)	0.97 (0.89, 1.04)
proline	1.04 (0.80, 1.29)	1.28 (1.00, 1.57) *	1.24 (1.00, 1.49) *	1.27 (1.02, 1.52) *
serine	0.98 (0.91, 1.04)	0.92 (0.85, 0.98)	1.08 (0.98, 1.18)	0.95 (0.84, 1.05)
arginine	0.86 (0.82, 0.91)	0.98 (0.84, 1.12)	1.08 (1.00, 1.15)	1.16 (0.96, 1.36)
**Gluconeogenic and ketogenic amino acids**				
phenylalanine	0.89 (0.83, 0.95) *	1.35 (1.20, 1.50) *	0.94 (0.90, 0.97)	1.07 (0.91, 1.21)
threonine	1.17 (1.05, 1.29)	0.92 (0.81, 1.02)	1.08 (0.92, 1.25)	1.09 (0.97, 1.21)
tryptophan	1.00 (0.69, 1.31)	1.43 (1.17, 1.69) *	1.22 (0.94, 1.50)	0.97 (0.77, 1.16)
tyrosine	0.87 (0.71, 1.04)	1.00 (0.87, 1.14)	1.08 (0.92, 1.25)	1.09 (0.97, 1.21)
**Ketogenic amino acids**				
lysine	0.82 (0.74, 0.90) *	0.65 (0.55, 0.74) *	0.90 (0.75, 1.06)	0.99(0.79, 1.19)

Data were expressed as mean fold difference (95% CI) of HFD + Leu and HFD + CR rats relative to their corresponding HFD control at week 24 and 32, respectively (*n* = 8). * *p* < 0.05 versus age-matched HFD controls. HFD: High-fat diet; CR: Calorie restriction.

**Table 4:** Fold difference of serum metabolites significantly changed in HFD rats relative to the corresponding ND controls.

Metabolites	24 weeks	32 weeks
**Amino acids derivatives**		
L-Kynurenine	0.72 (0.64, 0.79) *	1.09 (0.87, 1.31)
5-hydroxytryptophan	0.55 (0.44, 0.66) *	1.11 (0.93, 1.29) *
Spermidine	0.52 (0.48, 0.57) *	1.26 (1.10, 1.42)
2-hydroxyisobutyrate	0.86 (0.77, 0.95)	2.23 (1.87, 2.59) *
2-hydroxyglutarate	0.55 (0.47, 0.62) *	1.37 (1.20, 1.54) *
S-(adenosyl)-L-homocysteine	0.49 (0.41, 0.58) *	1.28 (0.96, 1.60)
4,6-dihydroxyquinoline	0.55 (0.49, 0.62) *	1.20 (0.93, 1.46)
Creatinine	0.43 (0.33, 0.54) *	1.19 (0.98, 1.40)
Creatine	0.41 (0.31, 0.52) *	1.34 (1.04, 1.64) *
Uridine	0.39 (0.25, 0.52) *	1.36 (1.17, 1.54) *
**Fatty acid metabolism**		
3-hydroxybutyric	0.77 (0.70, 0.85) *	1.92 (1.56, 2.27) *
Palmitic acid	3.92 (3.25, 4.59) *	1.53 (0.93, 2.12)
Acetylcarnitine	0.70 (0.64, 0.77) *	1.02 (0.84, 1.19)
Palmitoyl-L-carnitine	0.91 (0.75, 1.08)	1.48 (1.11, 1.86)
Carnitine	0.46 (0.38, 0.54) *	1.04 (0.68, 1.41)
**Phospholipids**		
Lysophosphatidylcholine (C18:1)	0.50 (0.43, 0.57) *	1.18 (0.99, 1.37)
Lysophosphatidylcholine (C16:1)	0.38 (0.34, 0.41) *	0.99 (0.82, 1.16)
Lysophosphatidylcholine (C16:0)	0.65 (0.58, 0.72) *	0.97 (0.83, 1.12)
Lysophosphatidylcholine (C20:4)	0.16 (0.12, 0.20) *	1.21 (0.94, 1.48)
**TCA cycle intermediates**		
Fumaric acid	0.82 (0.74, 0.89) *	1.12 (0.93, 1.31)
Malic acid	3.54 (2.81, 4.27) *	1.23 (1.07, 1.39) *
Citric acid	0.91 (0.80, 1.03)	1.22 (1.10, 1.34) *
a-Ketoglutaric acid	0.52 (0.45, 0.59) *	1.13 (0.98, 1.29)
**Bile acid metabolism**		
Ursodeoxycholic acid	0.28 (0.19, 0.38) *	1.15 (0.73, 1.56)
Glycochenodeoxycholic acid	0.32 (0.28, 0.35) *	0.73 (0.60, 0.86)
Glycocholic acid	0.71 (0.13, 1.28)	0.65 (0.22, 1.08)
**Purine metabolism**		
Xanthosine	0.71 (0.46, 0.96)	0.93 (0.71, 1.16)
Allantoin	0.42 (0.33, 0.50) *	1.00 (0.85, 1.14)
Uric acid	0.88 (0.77, 0.98)	1.10 (0.95, 1.25)
Xanthine	0.83 (0.56, 1.09)	1.00 (0.71, 1.29)

Data were expressed as mean fold difference (95% Confidence Interval) of HFD-fed rats relative to their corresponding ND controls at week 24 and 32, respectively (*n* = 8). * *p* < 0.05 versus age-matched ND controls. HFD: High fat diet; ND: Normal chow diet; TCA, tricarboxylic acid.

**Table 5:** Fold difference of serum metabolites significantly changed in HFD + CR or HFD + Leu rats relative to the corresponding HFD-fed controls.

	24 weeks		32 weeks	
Metabolites	HFD + CR	HFD + Leu	HFD + CR	HFD + Leu
**Amino acids derivatives**				
L-Kynurenine	1.30 (0.66, 1.95)	1.70 (1.23, 2.19) *	1.10 (0.43, 1.78)	1.10 (0.81, 1.39)
5-hydroxytryptophan	1.10 (0.96, 1.25)	1.58 (1.16, 1.92) *	1.29 (0.82, 1.76)	1.43 (1.07, 1.79) *
Spermidine	1.17 (1.02, 1.33) *	1.43 (1.26, 1.60) *	1.18 (1.04, 1.32) *	1.05 (0.89, 1.21)
2-hydroxyisobutyrate	0.90 (0.70, 1.10)	1.54 (1.26, 1.81) *	0.82 (0.56, 1.09)	0.67 (0.38, 0.95) *
2-hydroxyglutarate	0.88 (0.73, 1.03)	1.71 (1.50, 1.92) *	1.07 (0.86, 1.28)	1.01 (0.86, 1.16)
S-(adenosyl)-L-homocysteine	1.24 (0.87, 1.60)	1.57 (1.28, 1.86) *	1.51 (1.20, 1.83) *	1.34 (1.03, 1.66) *
4,6-dihydroxyquinoline	1.54 (1.21, 1.87) *	1.81 (1.56, 2.06) *	1.50 (1.17, 1.82) *	0.90(0.75, 1.05)
Creatinine	1.31 (1.19, 1.43) *	1.50 (1.36, 1.64) *	1.14 (0.91, 1.37)	1.16 (0.91, 1.40)
Creatine	0.91 (0.76, 1.06)	1.33 (0.99, 1.68)	1.04 (0.70, 1.37)	1.71 (1.34, 2.08) *
Uridine	1.32 (0.93, 1.72)	1.61 (1.37, 1.85*	1.02 (0.88, 1.17)	0.92 (0.68, 1.17)
**Fatty acid metabolism**				
3-hydroxybutyric	0.92 (0.71, 1.13)	1.36 (1.13, 1.59) *	0.92 (0.60, 1.24)	0.91 (0.48, 1.33)
Palmitic acid	1.28 (1.03, 1.53) *	1.81 (1.47, 2.15) *	1.55 (1.17, 1.94) *	0.54 (0.38, 0.70) *
Acetylcarnitine	1.12 (0.96, 1.30)	1.34 (1.21, 1.48) *	1.15 (0.92, 1.39)	0.87 (0.67, 1.07)
Palmitoyl-L-carnitine	1.17 (0.97, 1.37)	1.90 (1.54, 2.26) *	0.99 (0.59, 1.40)	0.49 (0.39, 0.58) *
Carnitine	1.29 (1.09, 1.48) *	1.86 (1.46, 2.26) *	1.28 (1.09, 1.47)	1.04 (0.93, 1.15)
**Phospholipids**				
Lysophosphatidylcholine (C18:1)	1.35 (1.13, 1.56) *	1.42 (1.19, 1.65) *	1.55 (1.19, 1.90) *	1.49 (1.14, 1.83) *
Lysophosphatidylcholine (C16:1)	1.40 (1.15, 1.64) *	1.42 (1.23, 1.60) *	1.41 (1.00, 1.83) *	0.91 (0.71, 1.10)
Lysophosphatidylcholine(C16:0)	1.20 (1.05, 1.36) *	1.34 (1.18, 1.49) *	1.31 (1.13, 1.48) *	1.21 (1.03, 1.40) *
Lysophosphatidylcholine (C20:4)	1.06 (0.87, 1.25)	1.04 (0.77, 1.30)	0.87 (0.50, 1.24)	3.82 (3.23, 4.41) *
**TCA cycle intermediates**				
Fumaric acid	1.09 (0.93, 1.24)	1.23 (1.08, 1.38) *	1.09 (0.96, 1.22)	0.82 (0.72, 0.93) *
Malic acid	0.87 (0.74, 1.00)	1.33 (1.11, 1.55) *	0.97 (0.48, 1.47)	0.15 (0.09, 0.22) *
Citric acid	1.02 (0.90, 1.15)	1.18 (0.97, 1.39)	0.94 (0.77, 1.11)	0.86 (0.74, 0.97) *
a-Ketoglutaric acid	0.95 (0.80, 1.09)	1.32 (1.13, 1.50) *	1.23 (1.00, 1.47)	1.05 (0.85, 1.25)
**Bile acid metabolism**				
Ursodeoxycholic acid	1.57 (0.96, 2.19)	2.58 (1.91, 3.24) *	1.37 (1.06, 1.68) *	1.35 (1.08, 1.61) *
Glycochenodeoxycholic acid	1.45 (1.19, 1.71) *	1.26 (0.92, 1.60)	1.63 (1.13, 2.13) *	1.43 (1.19, 1.66) *
Glycocholic acid	2.20 (1.04, 3.36) *	4.17 (0.60, 7.74)	3.17 (1.08, 5.27) *	3.76 (1.45, 6.07) *
**Purine metabolism**				
Xanthosine	0.90 (0.63, 1.18)	1.20 (0.77, 1.62)	1.49 (1.13, 1.85) *	1.05 (0.90, 1.19)
Allantoin	1.05 (0.86, 1.24)	1.33 (1.00, 1.66) *	1.27 (1.04, 1.50) *	1.41 (1.23, 1.59) *
Uric acid	0.91 (0.77, 1.05)	1.24 (1.07, 1.41) *	1.08 (0.93, 1.23)	0.90 (0.83, 0.96)
Xanthine	1.05 (0.55, 1.55)	1.26 (0.76, 1.76)	1.71(1.38, 2.02) *	1.12 (0.96, 1.27)

Data were expressed as mean fold difference (95% Confidence Interval) of HFD + Leu and HFD + CR rats relative to their corresponding HFD controls at week 24 and 32, respectively (*n* = 8). * *p* < 0.05 versus age-matched HFD controls. HFD: High fat diet; CR: Calorie restriction.

## Supplementary Materials

The following are available online at www.mdpi.com/2072-6643/9/6/565/s1.
