# Supplementary material for: Leucine Supplementation Differently Modulates Branched-Chain Amino Acid Catabolism, Mitochondrial Function and Metabolic Profiles at the Different Stage of Insulin Resistance in Rats on High-Fat Diet
Source: Nutrients. 2017 Jun 2;9(6):565. doi: 10.3390/nu9060565 (PMC5490544; doi:10.3390/nu9060565)
Supplement: Supplementary file 1 [file nutrients-09-00565-s001.pdf]

Supplementary data

Table S1. Primary antibodies used in this study.

rabbit anti-AKT (1:1,000 dilution; Cell Signaling Technology, Danvers, MA)
rabbit anti-phosphoSer473-AKT (1:1,000 dilution; Cell Signaling Technology, Danvers, MA)
rabbit anti-BCATm (1:250 dilution; Sangon Biotech Co., Ltd., Shanghai, China)
rabbit anti-BCKDHE1α (1:250 dilution; Sangon Biotech Co., Ltd., Shanghai, China)
rabbit anti-BCKDK (1:5,000 dilution; Abcam, Cambridge, MA)
MitoProfile Total OXPHOS Rodent WB antibody cocktail (1:250 dilution; Abcam, Cambridge, MA)
rabbit anti-GAPDH (1:1,000 dilution; Cell Signaling Technology, Danvers, MA)
rabbit anti-porin (1:1,000; Sangon Biotech Co., Ltd., Shanghai, China)

Table S2. Primer sequences used for real-time PCR.

**Gene**	**Primer (Forward)**	Primer (Reverse)
PGC-1α	GCCACTACAGACACCGC	CCTTTCAGACTCCCGCT
SIRT1	GAACCTCTGCCTCATCTAC	ATACTCGCCACCTAACCTA
β-globin	GGGGAAAGGTGAATGCTGATAATGT	ATGATAGCAGAGGCAGAGGACAGGT
COX II	GAAGTTGATAATCGGGTAG	CGGTTTGATGTGACTGTAG
TFAM	CTACAGAACAGCTACCCAAA	TTAAAATCCGCTTCATACAC
NRF-1	GACTCCCCTTCCTCGCCTG	CCTTTTCCGTTTCTTCCCT
GAPDH	GCAAGTTCAACGGCACAG	GCCAGTAGACTCCACGACAT

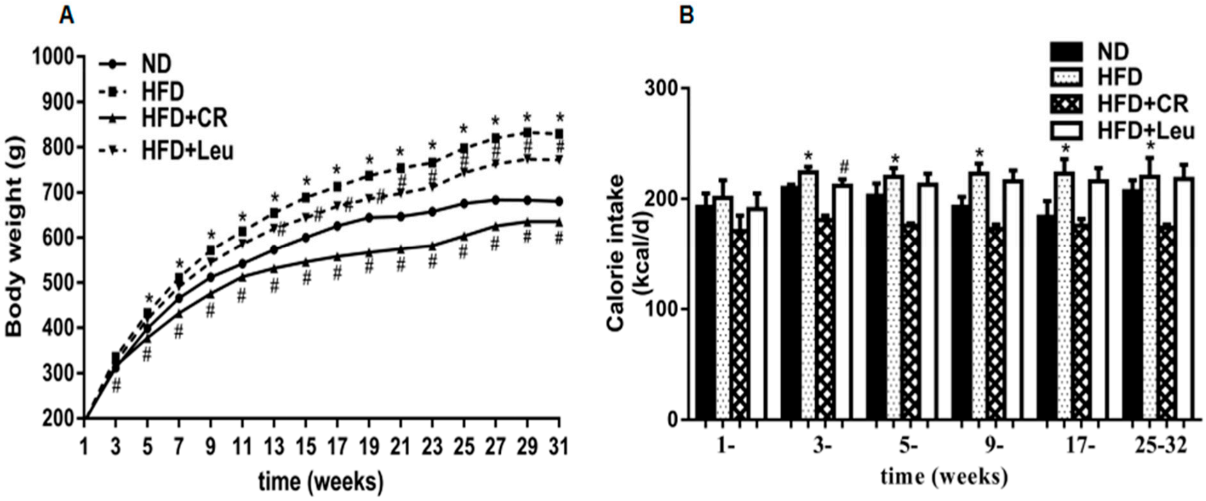

Figure S1. Effects of leucine supplementation on body weight and calorie intake in rats fed a HFD. (**A**) Body weights were monitored once per week. (**B**) Calorie intake was calculated based on the average amount of food ingested and the calorie content in the ND (3.8 kcal/g) or the HFD (4.79 kcal/g) (n=10). The results are presented as means±SD. **p*<0.05 compared with the ND-fed rats. #*p*<0.05 compared with the HFD-fed rats. &*p*<0.05 compared with the HFD+CR rats.
